# Urinary pneumococcal serotype detection among children with and without community-acquired pneumonia

**DOI:** 10.1186/s12879-025-11384-6

**Published:** 2025-08-07

**Authors:** Lilliam Ambroggio, Lindsay R. Grant, Jillian M. Cotter, Samuel R. Dominguez, Daniel Olson, Sara R. Saporta-Keating, Cody Bender, Mohammad Ali, Kathleen Grice, Gonzalo Lerner, Michael W. Pride, Ashley Miller, Maria J. Tort, Annaellis Vaughan, Elizabeth Temte, Alec Edid, Alejandro Cané, Kristina G. Hulten, Jill Stein, Bradford D. Gessner, Edwin J. Asturias

**Affiliations:** 1 Section of Emergency Medicine, Department of Pediatrics, Children’s Hospital Colorado, University of Colorado, Aurora, CO USA; 2 Section of Hospital Medicine, Department of Pediatrics, Children’s Hospital Colorado, University of Colorado, Aurora, CO USA; 3 Section of Infectious Diseases, Department of Pediatrics, Children’s Hospital Colorado, University of Colorado, Aurora, CO USA; 4 Pfizer, Inc., 500 Arcola Road, Collegeville, PA USA; 5 Infectious Diseases, Department of Pediatrics, Baylor College of Medicine, Houston, TX USA; 6 Vaccine Research and Development, Pfizer Vaccines, Pearl River, NY USA; 7 Department of Radiology, Children’s Hospital Colorado, University of Colorado, Aurora, CO USA

**Keywords:** Pediatrics, Pneumococcus, Serotype, Acute respiratory infections

## Abstract

**Background:**

Urinary antigen detection (UAD) assays can address diagnostic challenges with culture-based identification of *S. pneumoniae*. We aimed to evaluate the utility of Pfizer’s UAD1 and UAD2 assays for pneumococcal serotype surveillance in children with community acquired pneumonia (CAP) or upper respiratory tract infections (URI).

**Methods:**

From March 2021–December 2023, children 3 months to 5 years who presented to the Children’s Hospital Colorado Emergency Department with respiratory symptoms were enrolled as CAP or URI; healthy children served as controls. Nasal swabs were tested for pneumococcus by PCR. UAD assays identified pneumococcal serotypes from urine. Groups were compared using descriptive statistics.

**Results:**

We enrolled 407 controls, 202 with URI, and 280 with CAP. Positivity thresholds were set for all UAD serotypes. Pneumococcal nasal swab positivity was 23% for CAP, 17% for URI, and 3% in controls. Serotypes 3, 15B/C, 19 F, and 35B were frequent in nasal swabs in children with CAP or URI. UAD identified serotypes in 13% with CAP and 5% with URI. The most common urine serotypes were 19 F, 22 F, and 9 N. Among children with a nasal swab collected, 28/190 (15%) with CAP and 4/135 (3%) with URI had a positive UAD; **7** (3.7%) and 2 (1.5%), respectively, were the same serotype identified by nasal swab.

**Conclusions:**

UAD may be useful in differentiating pneumococcal serotypes in children with CAP and URI from controls. For most, serotypes identified in nasal swabs were not those identified by UAD suggesting UAD positivity was not due to pneumococcal carriage. The Pfizer UAD assay could be utilized for population monitoring of serotype distribution among children with CAP.

**Supplementary Information:**

The online version contains supplementary material available at 10.1186/s12879-025-11384-6.

## Introduction

Circulating pneumococcal serotypes in children with pneumonia have not been well documented due to multiple challenges including lack of bacteremia for most pneumonia (< 3%), and paucity of samples collected from other relevant sources (i.e. pleural effusions, lung parenchyma) [1, 2]. Urine based assays that detect pneumococcal antigens overcome these disadvantages and have been used to improve sensitivity while maintaining high specificity of detecting pneumococcus among adults with community acquired pneumonia (CAP). However, certain characteristics in the nasopharynx (NP) of children, such as frequent pneumococcal carriage and at a high inoculum, suggest the need to evaluate these assays among children. Currently, two platforms exist for detecting pneumococcus in the urine: (1) the pan-serotype pneumococcal urinary antigen test (PUAT, e.g., BinaxNOW) and (2) the serotype-specific urinary antigen detection (UAD) assays [3]. Both have been used in adults to detect and characterize cases of pneumococcal pneumonia [2]. However, the PUAT has limited specificity among young children in whom high prevalence and density of pneumococcal colonization yielded false-positive PUAT results [4, 5]. 

The Pfizer UAD assays (UAD1 and UAD2) are multiplex immunoassays that can simultaneously detect specific pneumococcal serotypes in urine. The UAD1 assay detects 13 serotypes (1, 3, 4, 5, 6 A, 6B, 7 F, 9 V, 14, 18 C, 19 A, 19 F, 23 F) [6] and the UAD2 assay detects an additional 11 serotypes (2, 8, 9 N, 10 A,11 A, 12 F, 15B, 17 F, 20, 22 F, 33 F) [7]. The serotype-specific UAD assays are intended for research only and not for diagnostic purposes due to the technical specifications of the assays. Specifically, the assays have been used to assess serotype distribution and to evaluate PCV13 efficacy or effectiveness among adults with radiologically confirmed CAP [3]. Using the UAD assays for similar purposes among children could address multiple data gaps, such as monitoring changes in pneumococcal serotype distribution in pneumonia following introduction of pneumococcal vaccines; assessing whether some serotypes are more commonly represented in non-bacteremic pneumonia compared to invasive disease; allowing for observational studies of pneumococcal vaccine effectiveness against serotype specific non-bacteremic pneumonia, which in turn might allow for expanded and more accurate regulatory labels; and more accurate determination of the overall burden of pneumococcus that in turn could more accurately populate economic models associated with this population [8]. The objective of the study was to determine the ability of Pfizer’s UAD assays to be used for pneumococcal serotype surveillance of children with CAP and URI.

## Methods

### Study design and population

Children were prospectively enrolled from Children’s Hospital Colorado (CHCO) and affiliated sites in the United States between March 30, 2021, and December 19, 2023, overlapping with the SARS-CoV-2 pandemic. Children were 3 months (90 days) to ≤ 5 years of age and enrolled into one of three cohorts: (1) the CAP cohort if presenting with two or more signs or symptoms of pneumonia and if either a chest radiograph had radiographic evidence of pneumonia, as read by the ED provider or on-call radiologist or antibiotics for treatment of pneumonia were prescribed, (2) the URI cohort if there was at least one upper respiratory symptom present, and (3) the control cohort if there was no evidence of a respiratory infection. Children were recruited consecutively in the ED by trained research assistants from 7am until midnight, 7 days a week; however, children who were recruited from other sites were consecutively recruited by trained research assistants from 9am to 4pm, 5 days a week. Children were excluded from participation in any cohort if they had received a dose of pneumococcal vaccine 30-days prior to enrollment, was hospitalized within 30 days of enrollment, or had a serious chronic disease (e.g., immunodeficiency; additional details provided in the Supplemental Materials).

### Ethics

The study was approved by the Colorado Multiple Institutional Review Board in accordance with the Declaration of Helsinki. Informed consent was obtained and required from all parents or legal guardians for their child’s participation in the study.

### Clinical data collection

Demographic characteristics and pre-visit disease course were recorded upon enrollment via a structured caregiver survey available in written format in English and Spanish and verbally translated via an interpreter when other languages were needed. Data on the management of the illness was abstracted from the electronic health record (EHR). All chest radiographs from children with CAP were reviewed by a pediatric radiologist (J.S.) using the World Health Organization standardized criteria [9]; radiographic confirmation was defined as the presence of alveolar consolidation or pleural effusion at enrollment. Pneumococcal vaccine history was obtained via the Colorado Immunization Information System (CIIS). Data were collected and managed using REDCap electronic data capture tools hosted at the University of Colorado [10]. Details on the data elements that were collected are available in the Supplemental Materials.

### Nasal swab collection and testing

A swab from the upper respiratory tract was obtained for detection of pneumococcus either by using residual standard of care-collected swabs or by collecting a protocol-specified swab. At the beginning of the study, nasopharyngeal swabs (NP) were obtained as standard of care from children for SARS-CoV-2 screening. Midway through the study, the hospital switched to using mid-turbinate swabs for viral testing as there was a shortage of clinical swabs for collection of NP samples; no other swab collection methods were modified. At that time, if a mid-turbinate swab was not obtained for clinical purposes, informed consent was obtained to collect a NP for research purposes. Participants could still be enrolled in the study even if an NP swab was not obtained or if the NP swab obtained for clinical reasons ended up not having enough residual to be tested. Both clinical and research swabs (collectively called “nasal swabs”) were inoculated into STGG media and tested in the Clinical Microbiology Laboratory at CHCO for pneumococcal colonization of the upper airway. If samples could not be tested immediately, they were frozen at −70 °C until the time of the assay. Pneumococcal colonization was determined based on a quantitative polymerase chain reaction (PCR) targeting three pneumococcal genes: *lytA*, *piaB*, and SP2020 [11, 12]. Positive pneumococcus was defined as at least two of the three gene targets having a cycle threshold (C_T_) value ≤ 40 in at least two replicate wells [12]. Quantification was based on the SP2020 C_T_ value measured against a standard curve with a reportable range between 1,000–10,000,000 genome copies/mL. Pneumococcal PCR-positive specimens were shipped to Baylor College of Medicine for serotyping if sufficient template DNA was available (defined as a *lyt*A PCR C_T_ < 32). Serotyping was performed by multiplex real-time PCR using the Centers for Disease Control and Prevention serotyping protocol that detects 64 serotypes or small serogroups [13]. Nasal swabs were also tested by the Biofire Respiratory Pathogen Panel (RPP) to detect the presence of respiratory viruses.

### Urine collection and testing

Up to 15 mL of urine was obtained for UAD testing. The PIPES (Piperazine-N, N’-bis (2-ethanesulfonic acid)) buffer was added to the urine sample within three hours of sample collection, samples were aliquoted, and frozen at −80 °C until testing by the UAD assay. Urine samples were batch shipped to Pfizer Vaccine Research Laboratory (Pearl River, New York) for UAD testing. The UAD assays are limit assays based on the Luminex platform where serotype-specific monoclonal antibodies coated individually onto spectrally unique microspheres are used to detect pneumococcal polysaccharide antigens secreted in the urine. The UAD assays produce a fluorescent signal which corresponds to an antigen concentration that is read off a standard curve. The antigen concentrations from the controls were used to define the positivity thresholds set per serotype using the pre-specified statistical method established for the original PCV13 efficacy trial among Dutch adults [6]. 

### Sterile site sample collection and testing

Blood and pleural fluid samples were collected as part of standard of care at the discretion of the treating physician and cultured in the CHCO microbiology lab using standard techniques. Pneumococcal isolates were collected, inoculated from pure culture growth into STGG media, and frozen at −80 °C for future testing. Frozen isolates were then shipped to the Infectious Diseases Research Laboratory at Texas Children’s Hospital (Houston, Texas) for confirmation of *S. pneumoniae* by optochin and bile solubility testing and serotyping by Quellung reaction [14]. 

### Statistical analysis

Count variables were described with median and interquartile range. Comparison across groups for statistical significance used the Median test or Mood’s Median test, as appropriate. Categorical variables were described using count and percentages and compared across groups using the Fisher’s Exact test or the Fisher-Freeman-Halton test as appropriate. Density values at the lower limit of detection (recorded as < 1,000 copies/mL) were imputed as 999 copies/mL. Positive samples were log10 transformed and categorized by density into one of three tertiles: low density (≤ 33%); medium density (34−65%); high density (≥ 66%). The analysis for this paper was generated using SAS release 9.04.01.

## Results

### Overview of the study population

A total of 889 children were enrolled: 407 healthy controls, 202 with URI, and 280 with CAP (Supplemental Table 1). Children with CAP were younger and were more likely to have a previous history of bronchospasm, previous hospitalization for a lower respiratory tract infection, or born prematurely (< 37 weeks) compared with children in control and URI groups. A higher proportion of children with CAP received antibiotics during the 14 days prior to enrollment or were using corticosteroids at enrollment (Table 1). Among 280 children with CAP, 132 (47.1%) children received antibiotics in ED or during the heath care encounter (most commonly amoxicillin [*n* = 47, 16.8%]), and 94 (33.6%) required ICU care. Among children with CAP, 82.5% (*n* = 231) had a radiographically confirmed pneumonia, while the remaining 17.5% (*n* = 49) had an infiltrate present that did not meet the radiographically confirmed definition (Table 1).

**Table 1:** Demographic and clinical characteristics of the enrolled children

	Controls (*N* = 407)	URI (*N* = 202)	CAP (*N* = 280)	*p*-value^a^
**Demographic characteristics**				
Age, median (IQR)	48.6 (28.6, 60.6)	43.5 (25.6, 55.3)	34.0 (18.2, 54.5)	< 0.01
Female sex, n (%)	165 (40.5)	82 (40.6)	115 (41.1)	0.99
Race, n (%)				
American Indian or Alaska Native	8 (2)	9 (4.5)	10 (3.6)	0.17
Asian	24 (5.9)	9 (4.5)	19 (6.8)	0.59
Black	57 (14)	39 (19.3)	33 (11.8)	0.07
Middle Eastern	4 (1)	3 (1.5)	1 (0.4)	0.45
Native Hawaiian or Pacific Islander	3 (0.7)	1 (0.5)	6 (2.1)	0.21
White	301 (74)	148 (73.3)	209 (74.6)	0.94
Other	25 (6.1)	15 (7.4)	18 (6.4)	0.83
Missing	27 (6.6)	9 (4.5)	17 (6.1)	0.57
Ethnicity, n (%)				
Hispanic	136 (33.4)	95 (47)	98 (35)	0.01
Non-Hispanic	256 (62.9)	104 (51.5)	173 (61.8)	0.02
Not recorded	15 (3.7)	3 (1.5)	9 (3.2)	0.32
Previous medical history of asthma, n (%)	27 (6.6)	41 (20.3)	67 (23.9)	< 0.01
**Clinical Characteristics**				
Symptoms within 24 h, n (%)				
Fever (≥ 100.4 °F)	5 (1.2)	104 (51.7)	143 (52.2)	< 0.01
Cough	14 (3.5)	177 (88.9)	264 (96)	< 0.01
Difficulty breathing	0 (0)	121 (60.5)	236 (85.5)	< 0.01
Vomiting	45 (11.2)	82 (40.6)	83 (30.2)	< 0.01
Decreased oral fluid intake	32 (8)	96 (47.5)	156 (56.7)	< 0.01
Runny nose or congestion	22 (5.5)	175 (87.1)	247 (89.5)	< 0.01
Diarrhea	14 (3.5)	28 (14.1)	59 (21.5)	< 0.01
Chest pain	1 (0.3%)	27 (13.4)	53 (19.7)	< 0.01
Wheezing	0 (0)	102 (50.7)	145(52.5)	< 0.01
Received antibiotics received in the past 14 days, n (%)	22 (5.4)	15 (7.4)	83 (29.6)	< 0.01
Currently using corticosteroid, n (%)	8 (2)	35 (17.3)	68 (24.3)	< 0.01
Received any pneumococcal conjugate vaccine doses, n (%)	363 (89.2)	179 (88.6)	257 (91.8)	0.98
ED length of stay, median (IQR)	3.6 (2.5,6.3)	4.5 (3.3,6.4)	5.5 (4.2,9.0)	< 0.01
Received supplemental oxygen in the ED, n (%)	27 (11.8)	91 (45)	261 (93.9)	< 0.01
Received antibiotics in the ED or during encounter, n (%)	28 (12.3)	11 (5.4)	132 (47.3)	< 0.01
Lowest SpO_2_ at enrollment, median (IQR)	97.0 (94.0, 98.0)	94.0 (91.0, 96.0)	88.0 (84.0, 92.0)	< 0.01
Highest respiratory rate, median (breaths per min) (IQR)	26.0 (24.0, 32.0)	39.5 (28.0, 48.0)	52.0 (44.0, 62.0)	< 0.01
Encounter disposition, n (%)				
Discharged home from ED	138 (33.9)	132 (65.3)	13 (4.6)	< 0.01
Admitted to hospital from ED	63 (15.5)	70 (34.7)	262 (93.6)	< 0.01
Admitted to ICU	6 (1.5)	13 (6.4)	94 (33.6)	< 0.01
Hospitalization length of stay, median (days) (IQR)	2.0 (0.9, 4.5)	1.7 (1.0, 2.9)	4.2 (2.6, 6.7)	< 0.01
CXR obtained, n (%)	N/A	50 (24.8)	276 (98.6)	< 0.01
Overall CXR impression, n (%)^b^				
Normal	N/A	16 (7.9)	0	< 0.01
Definite atelectasis	N/A	19 (9.4)	8 (2.9)	< 0.01
Probable atelectasis	N/A	11 (5.4)	73 (26.1)	< 0.01
Atelectasis or pneumonia	N/A	3 (1.5)	101 (36.1)	< 0.01
Probable pneumonia	N/A	0	53 (18.9)	< 0.01
Definite pneumonia	N/A	1 (0.5)	41 (14.6)	< 0.01
Radiographically confirmed pneumonia, n (%)	N/A	4 (2)	231 (82.5)	< 0.01

CAP = community acquired pneumonia, CXR = chest x-ray, ED = emergency department, ICU = intensive care unit, IQR = interquartile range, N/A = not applicable, URI = upper respiratory infection

^a^ p-values for medians were calculated using the Mood’s Median test, all others using the Fisher-Freeman-Halton test

^b^ Chest radiographs were read by a pediatric radiologist using a standardized case report form using guidelines established by the World Health Organization working group {Cherian, 2005 #1141}

### Pneumococcal detection by UAD and from sterile sites

Urine samples were obtained from 405/407 (99.5%) controls and from all children with URI (*n* = 202) or CAP (*n* = 280). Positivity thresholds were set based on the control urine samples for all 24 serotypes covered by the UAD assays. In the overall population, UAD positivity was 5% (*n* = 10) and 13% (*n* = 36) among children with URI and CAP, respectively (Table 2). No difference was found in the UAD positivity between children with CAP who did and who did not meet the radiographically confirmed pneumonia definition (13% (30/231) vs. 12% (6/49). The most common serotypes detected by the UAD assays were 19 F, 22 F, and 9 N (Supplemental Table 2). Of the 14 children with CAP who had a positive sterile site culture for pneumococcus, 10 were from pleural fluid only, 3 were from blood culture, and one was from both pleural fluid and blood. Only one isolate (from blood) was retained for serotyping (19 F), which was also detected by the UAD assay (also from the nasal swab).

**Table 2:** UAD results among children with URI and CAP

	URI (*N* = 202)	CAP (*N* = 280)	RAD + CAP (*N* = 231)	Non-RAD + CAP (*N* = 49)
Urine specimen collected, n (%)​	202 (100)	280 (100)	231 (100)	49 (100)
Sterile site specimen collected, n (%)	0 (0)	51 (18)	44 (19)	7 (14)
*S. pneumoniae* positive, n (%)^a^	10 (5)	42 (15)	36 (16)	6 (12)
Positive by UAD1 or UAD2​	10 (5)	36 (13)	30 (13)	6 (12)
Positive by culture​ of sterile site	0	10 (4)	10 (4)	0 (0)
Positive with serotype information^b^	10 (5)	36 (13)	30 (13)	6 (12)
Top 5 serotypes identified by UAD, n (%)				
19 F	2 (1)	6 (2)	5 (2)	1 (2)
22 F	1 (0.5)	6 (2)	5 (2)	1 (2)
9 N	1 (0.5)	6 (2)	3 (1)	3 (6)
14	0 (0)	5 (2)	5 (2)	0 (0)
15B/C	2 (1)	2 (1)	2 (1)	0 (0)

CAP = community acquired pneumonia, RAD + CAP = radiologically-confirmed pneumonia, UAD1 = urinary antigen detection assay 1 (for detection of serotypes 1, 3, 4, 5, 6 A, 6B, 7 F, 9 V, 14, 18 C, 19 A, 19 F, 23 F), UAD2 = urinary antigen detection assay 2 (for detection of serotypes 2, 8, 9 N, 10 A, 11 A, 12 F, 15B, 17 F, 20, 22 F, 33 F), URI = upper respiratory infection

^a^ *S. pneumoniae* positive indicates that the sample was positive by either UAD1, UAD2, or by culture of sterile site. The categories are not mutually exclusive

^b^Among the pneumococci isolated from sterile sites of children with CAP, only one was serotyped. It serotyped as 19 F, which was the same serotype detected in the child’s urine by the UAD1 assay

### *S. pneumoniae* and viral pathogen detection from nasal swabs

Nasal swabs for *S. pneumoniae* testing were obtained from 190/280 (68%) children with CAP, 135/202 (67%) children with URI, and 85/407 (21%) control children. Children with CAP were more likely to have pneumococcus present (*n* = 65, 34%) when compared with the URI group (*n* = 35, 26%) or controls (*n* = 12, 14%). The median log10 pneumococcal density was higher among children with URI (4.1; Interquartile range [IQR]: 3.2, 4.8) than children with CAP (3.9; IQR: 3.3, 4.8) and controls (3.6; IQR: 3.2, 4.4), and higher in children aged 3-<12 months compared with children aged 36–72 months, across cohorts. Among children CAP, the most identified pneumococcal serotypes in carriage were 19 F (4.2%), 3 and 35B (2.6% each) while for children with URI the most common were 19 F, 15B/C and 35B (3% each) **(**Table 3**).**

**Table 3:** Pneumococcal detection and serotype distribution from nasal swabs (NP or MT) among children by cohort

	Controls (*N* = 407)	URI (*N* = 202)	CAP (*N* = 280)
**Type of nasal swab specimen collected** ***n*** **(%)**			
NP specimen collected	52 (13)	96 (48)	63 (23)
MT specimen collected	34 (8)	39 (19)	154 (55)
Either NP or MT specimen collected	85 (21)	135 (67)	190 (68)
*S. pneumoniae* positive, n (%)​			
Positive by NP​	8 (15)	26 (27)	20 (32)
Positive by MT	4 (12)	9 (23)	52 (34)
Positive by either NP or MT	12 (14)	35 (26)	65 (34)
*S. pneumoniae* density, log10 median (IQR)	3.6 (3.2, 4.4)	4.1 (3.2, 4.8)	3.9 (3.3, 4.8)
Total with a serotype identified, n (%)	11/85 (13)	34/135 (25)	55/190 (29)
Serotype identified, n (%)			
3	1 (1.2)	3 (2.2)	5 (2.6)
6	1 (1.2)	0 (0)	0 (0)
7 C	0 (0)	1 (0.7)	0 (0)
7 F	0 (0)	0 (0)	1 (0.5)
8	0 (0)	0 (0)	4 (2.1)
11 A	1 (1.2)	0 (0)	4 (2.1)
13	0 (0)	0 (0)	1 (0.5)
15 A	0 (0)	0 (0)	4 (2.1)
15B/C	0 (0)	4 (3)	4 (2.1)
17 F	1 (1.2)	1 (0.7)	0 (0)
16/16F^a^	0 (0)	1 (0.7)	1 (0.5)
19 A	0 (0)	0 (0)	2 (1.1)
19 F	0 (0)	4 (3)	8 (4.2)
20	1 (1.2)	0 (0)	1 (0.5)
22 F	0 (0)	1 (0.7)	2 (1.1)
23 F	0 (0)	0 (0)	1 (0.5)
31	0 (0)	1 (0.7)	0 (0)
35B	0 (0)	4 (3)	5 (2.6)
35 F	0 (0)	0 (0)	2 (1.1)
38	0 (0)	0 (0)	1 (0.5)
Not serotyped	7 (8.2)	15 (11.1)	14 (7.4)

CAP = community acquired pneumonia, IQR = interquartile range, MT = mid-turbinate, NP = nasopharyngeal, URI = upper respiratory infection

^a^ The URI case typed as serogroup 16

Respiratory pathogen panel testing occurred in 204/280 (73%) children with CAP, 145/202 (72%) children with URI, and in 23/407 (6%) controls. A virus was detected in close to 90% of children with URI or CAP, but in only 26% of controls. The most common virus detected was rhinovirus among children with URI and controls, while RSV was most common among children with CAP (Table 4). Parainfluenza and RSV were common among children with URI (16% and 19%, respectively). Parainfluenza and rhinovirus were common among children with CAP (13% and 21%, respectively), along with human metapneumovirus (18%; Table 4). *S. pneumoniae* detection and density from the nasal swab were similar for URI and CAP children with and without a viral pathogen detected (Supplemental Table 3).

**Table 4:** Respiratory virus detection among children by cohort

	Controls (*N* = 407)	URI (*N* = 202)​	CAP (*N* = 280)​
Respiratory Pathogen Panel testing performed, n (%)	23 (6)	145 (72)	204 (73)
Any virus detected	6 (26)	130 (90)	176 (86)
**Type of virus detected**,** n (%)**			
Adenovirus	1 (4)	13 (9)	23 (1)
Any Coronavirus (excluding SARS-CoV-2)	2 (9)	5 (3)	9 (4)
Coronavirus SARS-CoV-2	0 (0)	5 (3)	5 (2)
Human Metapneumovirus	0 (0)	4 (3)	37 (18)
Human rhinovirus/enterovirus	3 (13)	81 (56)	58 (28)
Any influenza virus	0 (0)	4 (3)	7 (3)
Any parainfluenza virus	1 (4)	23 (16)	27 (13)
RSV	0 (0)	27 (19)	69 (34)

CAP = community acquired pneumonia, RSV = Respiratory Syncytial Virus, URI = upper respiratory infection

### Nasal swab among UAD positive and negative children

Children in the URI or CAP cohorts with a UAD positive urine sample were more likely to have a nasal swab positive for pneumococcus than UAD negative children (Table 5). All (3/3) UAD positive children with URI had nasal swab pneumococcal densities in the high tertile group (log10 transformed value range: 4.44–5.99) whereas 28% (9/32) of UAD negative children had nasal swab pneumococcal densities in the high tertile group (*p* = 0.03). Among children with CAP, the percentages with nasal swab pneumococcal densities in the higher tertile group were 53% (8/15) among UAD positives and 30% (14/47) among UAD negatives (*p* = 0.13) (Table 5). Most children with a positive nasal swab were not UAD positive for the same serotype (Table 6). In both URI and CAP, the same percentage of children who were serotype 19 F positive and negative by UAD were colonized with 19 F (Table 6).

**Table 5:** *S pneumoniae* nasal swab results among UAD positive and negative children by cohort

	URI (*N* = 135)			CAP (*N* = 190)		
	UAD positive (*N* = 4)	UAD negative (*N* = 131)	*p*-value^a^	UAD positive (*N* = 28)	UAD negative (*N* = 162)	*p*-value^a^
*S pneumoniae* positive nasal swab, n (%)	3 (75)	32 (24)	0.05	15 (54)	50 (31)	0.03
Nasal swab density log10 median (IQR)	5.2 (4.6, 5.4)	4.0 (3.2, 4.6)	0.07	4.7 (3.0, 5.1)	3.9 (3.3, 4.5)	0.77
Nasal swab density category (log10 density range)^b^, n (%)						
High (4.44–5.99)	3 (100)	9 (28)	0.03	8 (53)	14 (30)^c^	0.13
Medium (3.53–4.43)	0 (0)	13 (41)	0.28	2 (13)	17 (36)^c^	0.12
Low (3.00-3.51)	0 (0)	10 (31)	0.54	5 (33)	16 (34)^c^	1.00

CAP = community acquired pneumonia, IQR = interquartile range, UAD = urinary antigen detection assay, URI = upper respiratory infection

^a^ The p-values were derived using Fisher’s exact test when comparing proportions and the median test when comparing the medians

^b^ The SP2020 rtPCR results (copies/mL) from positive nasal swabs were log10 transformed and categorized into one of three density tertile groups: low density (≤ 33%); medium density (34%−65%); high density (≥ 66%)

^c^ The denominator for the CAP UAD negative group is 47 because three of these children were missing density results

**Table 6:** Matching and non-matching serotype pairs from nasal swabs and the UAD assay

	*N* ^a^	Nasal swab positive and UAD positive		Nasal swab positive and UAD negative	
		*n* (%)	Log10 Median (IQR)	*n* (%)	Log10 Median (IQR)
**URI cohort**					
Serotype					
3	3	0 (0)	–	3 (100)	3.7 (3.1, 4.3)
15B/C	4	0 (0)	–	4 (100)	4.9 (4.4, 5.3)
17 F	1	0 (0)	–	1 (100)	3.7
19 F	4	2 (50)	4.9 (4.6, 5.2)	2 (50)	4.6 (3.3, 5.9)
22 F	1	0 (0)	–	1 (100)	4.2
**CAP cohort**					
Serotype					
3	5	0 (0)	–	5 (100)	3.7 (3.6, 3.8)
7 F	1	0 (0)	--	1 (100)	4.0
8	3	1 (33)	4.7	2 (67)	4.0 (3.9, 4.1)
11 A	4	0 (0)	–	4 (100)	4.4 (3.8, 5.1)
15B/C	4	0 (0)	–	4 (100)	5.1 (4.7, 5.5)
19 A	2	0 (0)	–	2 (100)	5.0 (4.2, 5.9)
19 F	8	4 (50)	4.9 (4.4, 5.5)	4 (50)	3.8 (3.0, 4.6)
20	1	0 (0)	–	1 (100)	3.4
22 F	2	2 (100)	3.0 (3.0, 3.0)	0 (0)	–
23 F	1	0 (0)	–	1 (100)	4.1

CAP = community acquired pneumonia, IQR = interquartile range, UAD = urinary antigen detection, URI = upper respiratory infection

^a^ N = Total children included in this analysis based on nasal swab testing that was PCR positive for the serotype and having a urine sample tested by UAD

## Discussion

Although previous studies have used the UAD assays to identify serotypes causing CAP in adults globally and in children in low-income settings, this was the first pediatric study conducted in a high-income country to assess detection of pneumococcal serotypes in urine among children with CAP using Pfizer’s serotype-specific UAD assays. After using control urine samples to determine positivity thresholds for all 24 serotypes, UAD positivity was 5% and 13% of children with URI and CAP, respectively. Nasal swab positivity for pneumococcus was associated with UAD positivity; however, the pneumococcal serotype from nasal swabs usually differed from that identified by the UAD assay, indicating that positive UAD results were not due to carriage. These findings suggest that Pfizer’s UAD assays can detect pneumococcal serotypes in children causing CAP, including those cases that are nonbacteremic. In the future, Pfizer’s UAD assays might also be used to estimate PCV effectiveness among children, as was done previously for studies conducted among adults [15]. 

This study also detected pneumococcal serotypes in the urine of children with URI, which is less commonly considered to be associated with signs and symptoms of pneumococcal infection compared with CAP. However, two lines of evidence support that pneumococcus could be the etiology of URI. Firstly, among Finnish children who participated in the FinIP RCT and STEPS study, PCV10 prevented 12% (95% CI: 2–22%) of any respiratory tract infections (defined as rhinitis, cough, or wheezing with or without a fever).{Karppinen, 2019 #1855} Secondly, experimental inoculation with serotype 3 pneumococci in the nasopharynx of adult volunteers caused sore throat in 83% of participants.{Robinson, 2022 #1856} Based on these data, it is reasonable to speculate that pneumococcus could have an etiologic role in causing URI; however, it is unclear how much pneumococcal antigen in the urine triggers a UAD positive result.

A previous pediatric study using the Pfizer UADs conducted in Burkina Faso also found that UAD could distinguish pneumococcal pneumonia from controls, but unlike our study did not include data on quantitative density, respiratory virus testing, or pneumococcal serotyping of nasal swabs [16]. In Burkina Faso, children with radiologically confirmed pneumonia were more likely to have a positive UAD assay (28.7% for RAD + CAP vs. 9.3% for non-RAD + CAP) while in our study these values were equal (13% vs. 12%). This difference, as well as the high proportion of UAD positivity in Burkina Faso among children with radiologically confirmed pneumonia, could reflect later presentation for care of Burkinabe children and hence larger pulmonary infiltrates, later and less effective antibiotic use for pneumococcal pneumonia in Burkinabe children, differences in serotype distribution, or differences in radiologic interpretation across settings.

Pediatric studies that used the PUAT revealed that the presence of pneumococcal colonization, likely at higher densities, yielded false positive urine antigen test results [3, 5]. In our study, UAD positivity was also more common among children with pneumococcus detected from a nasal swab, but with little concurrence between the colonizing and infecting serotypes. While pneumococcal carriage is considered a prerequisite for pneumococcal disease, these data suggest that the UAD can identify the serotypes causing pneumonia, which were typically different than those colonizing the upper respiratory tract. This could be accounted for by several factors including (1) if the serotypes causing CAP have a shorter duration of carriage and the serotypes less likely to cause CAP have a longer duration colonization and (2) if the serotypes causing CAP are more invasive than those colonizing the upper respiratory tract. Furthermore, the serotypes colonizing at the highest densities did not necessarily correlate with UAD positivity.

The five most prevalent serotypes detected by the UAD were 19 F, 22 F, 9 N, 14, and 15B/C. Most of these serotypes were also found among children with invasive pneumococcal disease (IPD) that was identified by the US Centers for Disease Control and Prevention Active Bacterial Core Surveillance (ABCs) during the same time, which partially overlapped with the SARS-CoV-2 pandemic [17]. Based on IPD surveillance among US children age < 5 years, serotypes 15B, 15 C, 19 F, and 22 F were among the top 10 serotypes causing IPD during 2019–2021, ranging from 4 to 9% of serotyped IPD. Serotype 9 N accounted for ~ 1% of IPD, and there were no cases of serotype 14 IPD among this age group in 2019–2021. Serotype 14 is covered by PCV7 and PCV13, which were introduced in 2001 and 2010 in the US pediatric immunization schedule. Detection of this serotype among children with CAP could indicate persistent circulation and transmission due to lower vaccine coverage that impacted many states during the pandemic and post-pandemic period.

Our study had several limitations. First, less than half of the controls and around two-thirds of children with URI and CAP had a nasal swab collected due to specimen collection restrictions during the acute phase of the COVID-19 pandemic. Consequently, understanding the implications of pneumococcal colonization on setting the UAD positivity thresholds could be incomplete. Second, MT swab collection replaced NP swab collection mid-way through the study and the sensitivity of pneumococcal detection from MT swabs is unknown; however, the percentage of MT and NP swabs that were pneumococcal positive was similar. In addition to this, the sensitivity of the laboratory methods used for pneumococcal detection and serotyping is unknown. Therefore, some low-density colonization episodes and serotypes could have been missed. These episodes are less likely to be associated with UAD positivity. Third, the small sample of children with CAP that are UAD positive, and the limited number of serotypes covered by the UAD assays (24 serotypes total) make it difficult to infer if the serotype distributions of CAP and IPD differ. Fourth, it is possible that children with CAP were more likely to be clinically severe and/or dehydrated during their ED encounter, and therefore were in the ED longer to receive fluids or care compared with children with URI. Fifth, the study period overlapped with the SARS-CoV-2 pandemic when pneumococcal serotype epidemiology had been perturbed, which could have affected circulating serotypes and pneumococcal disease trends. Sixth, the sensitivity and specificity of the UAD assays for bacteremic pneumonia have been established for adults but not for children in whom factors such as colonization frequency, bacterial load, antibiotic pretreatment could differ. Seventh, the sensitivity and specificity of the UAD against non-bacteremic pneumonia has not been determined, including for adults due to lack of a gold standard test for detecting *S. pneumoniae* from non-bacteremic pneumonia. Existing data suggest limited sensitivity based on estimates of PCV13 effectiveness against all cause pneumonia [8, 18]. Therefore, data from UAD assays should not be used to estimate serotype-specific disease burden (i.e., incidence).

The Pfizer UAD assay distinguished children with CAP from children with URI and from controls based on the criteria of setting positivity thresholds for each serotype. Moreover, UAD positivity among children with CAP did not appear to be due to pneumococcal carriage. UAD also identified an important fraction of disease due to pneumococcal serotypes detected by UAD among children with CAP (either radiographically confirmed or not) that was not otherwise identified by culture or detection of pneumococcus from blood or other sterile site specimens. These data provide insights into the potential distribution of serotypes among children in the US causing non-bacteremic CAP and thus the fraction potentially preventable by pneumococcal vaccines. As in adults, the UAD assays could become valuable tools for use in pediatric serotype epidemiology studies and in studies estimating PCV effectiveness and rate reductions for CAP following public health pneumococcal vaccine introductions.

## Supplementary Information

Supplementary Material 1

## Data Availability

Data is available by request and with institutional agreements.

## Abbreviations

AVAPS: Average volume-assured pressure support
BiPAP: Bilevel positive airway pressure
CAP: Community acquired pneumonia
CDC: Centers disease control and prevention
CHCO: Children’s Hospital Colorado
CIIS: Colorado immunization information system
COVID-19: Corona virus disease 2019
CRP: C-reactive protein
CPAP: Continuous positive airway pressure
C_T_: Cycle threshold
ED: Emergency Department
eCRF: Electronic case report form
EMR: Electronic medical record
HIPAA: Health insurance portability and accountability act
ICD: Informed consent document
ICU: Intensive care unit
IRB: Institutional review board
IPD: Invasive pneumococcal disease
mAb: Monoclonal antibody
MT: Mid-turbinate
N/A: Not applicable
NP: Nasopharynx
PCR: Polymerase chain reaction
PCT: Procalcitonin
PCV: Pneumococcal conjugate vaccine
PIPES: Piperazine-N, N’-bis(2-ethanesulfonic acid)
RPP: Respiratory pathogen panel
RSV: Respiratory syncytial virus
SAE: Serious adverse event
SARS-CoV-2: Severe acute respiratory syndrome coronavirus 2
UAD: Urine antigen diagnostic test
UCD: University of Colorado Denver
URI: Upper respiratory infection
U.S.: United States

## References

[1] Neuman MI, Hall M, Lipsett SC, et al. Utility of blood culture among children hospitalized with community-acquired pneumonia. Pediatrics. 2017. 10.1542/peds.2017-1013. 28835382 10.1542/peds.2017-1013 PMC5574722

[2] Bhat N, O’Brien KL, Karron RA, Driscoll AJ, Murdoch DR, Pneumonia Methods Working G. Use and evaluation of molecular diagnostics for pneumonia etiology studies. Clin Infect Dis. 2012;54(Suppl 2):S153-8. 10.1093/cid/cir1060. 22403230 10.1093/cid/cir1060 PMC3297547

[3] Isturiz R, Grant L, Gray S, et al. Expanded analysis of 20 pneumococcal serotypes associated with radiographically confirmed community-acquired pneumonia in hospitalized US adults. Clin Infect Dis. 2021;73(7):1216–22. 10.1093/cid/ciab375. 33982098 10.1093/cid/ciab375 PMC8492118

[4] Michelow IC, Lozano J, Olsen K, et al. Diagnosis of Streptococcus pneumoniae lower respiratory infection in hospitalized children by culture, polymerase chain reaction, serological testing, and urinary antigen detection. Clin Infect Dis. 2002;34(1):E1-11. 10.1086/324358. 11731965 10.1086/324358

[5] Adegbola RA, Obaro SK, Biney E, Greenwood BM. Evaluation of Binax now Streptococcus pneumoniae urinary antigen test in children in a community with a high carriage rate of pneumococcus. Pediatr Infect Dis J. 2001;20(7):718–9. 10.1097/00006454-200107000-00018. 11465850 10.1097/00006454-200107000-00018

[6] Pride MW, Huijts SM, Wu K, et al. Validation of an immunodiagnostic assay for detection of 13 Streptococcus pneumoniae serotype-specific polysaccharides in human urine. Clin Vaccine Immunol. 2012;19(8):1131–41. 10.1128/CVI.00064-12. 22675155 10.1128/CVI.00064-12 PMC3416073

[7] Kalina WV, Souza V, Wu K, et al. Qualification and clinical validation of an immunodiagnostic assay for detecting 11 additional Streptococcus pneumoniae serotype-specific polysaccharides in human urine. Clin Infect Dis. 2020;71(9):e430-8. 10.1093/cid/ciaa158. 32072165 10.1093/cid/ciaa158 PMC7713672

[8] Bonten MJ, Huijts SM, Bolkenbaas M, et al. Polysaccharide conjugate vaccine against Pneumococcal pneumonia in adults. N Engl J Med. 2015;372(12):1114–25. 10.1056/NEJMoa1408544. 25785969 10.1056/NEJMoa1408544

[9] Cherian T, Mulholland EK, Carlin JB, et al. Standardized interpretation of paediatric chest radiographs for the diagnosis of pneumonia in epidemiological studies. Bull World Health Organ. 2005;83(5):353–9. 15976876 PMC2626240

[10] Harris PA, Taylor R, Thielke R, Payne J, Gonzalez N, Conde JG. Research electronic data capture (REDCap)--a metadata-driven methodology and workflow process for providing translational research informatics support. J Biomed Inform. 2009;42(2):377–81. 10.1016/j.jbi.2008.08.010. 18929686 10.1016/j.jbi.2008.08.010 PMC2700030

[11] Carvalho Mda G, Tondella ML, McCaustland K, et al. Evaluation and improvement of real-time PCR assays targeting lyta, ply, and psaA genes for detection of pneumococcal DNA. J Clin Microbiol. 2007;45(8):2460–6. 10.1128/JCM.02498-06. 17537936 10.1128/JCM.02498-06 PMC1951257

[12] Butler M, Breazeale G, Mwangi E, et al. Development and validation of a multiplex real-time PCR assay for detection and quantification of Streptococcus pneumoniae in pediatric respiratory samples. Microbiol Spectr. 2023;11(6):e0211823. 10.1128/spectrum.02118-23. 37937989 10.1128/spectrum.02118-23 PMC10715132

[13] Velusamy S, Tran T, Mongkolrattanothai T, Walker H, McGee L, Beall B. Expanded sequential quadriplex real-time polymerase chain reaction (PCR) for identifying pneumococcal serotypes, penicillin susceptibility, and resistance markers. Diagn Microbiol Infect Dis. 2020;97(2):115037. 10.1016/j.diagmicrobio.2020.115037. 32265073 10.1016/j.diagmicrobio.2020.115037

[14] Wasilauskas BL, Hampton KD. An analysis of *Streptococcus pneumoniae* identification using biochemical and serological procedures. Diagn Microbiol Infect Dis. 1984;2(4):301–7. 10.1016/0732-8893(84)90061-0. 6488747 10.1016/0732-8893(84)90061-0

[15] McLaughlin JM, Jiang Q, Isturiz RE, et al. Effectiveness of 13-valent pneumococcal conjugate vaccine against hospitalization for community-acquired pneumonia in older US adults: a test-negative design. Clin Infect Dis. 2018;67(10):1498–506. 10.1093/cid/ciy312. 29790925 10.1093/cid/ciy312 PMC6206101

[16] Bountogo M, Sanogo B, Pride MW, et al. Application of a Pneumococcal Serotype-specific urinary antigen detection test for identification of pediatric pneumonia in Burkina Faso. Pediatr Infect Dis J. 2021;40(5):418–25. 10.1097/INF.0000000000003065. 33464020 10.1097/INF.0000000000003065

[17] Centers for Disease Control and Prevention C. Active Bacterial Core (ABCs) Surveillance. Accessed 12/9/2024. 2024. https://www.cdc.gov/abcs/methodology/index.html#cdc_data_surveillance_section_7-case-ascertainment

[18] Gessner BD, Jiang Q, Van Werkhoven CH, et al. A public health evaluation of 13-valent pneumococcal conjugate vaccine impact on adult disease outcomes from a randomized clinical trial in the Netherlands. Vaccine. 2019;37(38):5777–87. 10.1016/j.vaccine.2018.05.097. 29861177 10.1016/j.vaccine.2018.05.097

