# Supplementary material for: Urinary pneumococcal serotype detection among children with and without community-acquired pneumonia
Source: BMC Infect Dis. 2025 Aug 7;25:995. doi: 10.1186/s12879-025-11384-6 (PMC12329884; doi:10.1186/s12879-025-11384-6)
Supplement: Supplementary file 1 — Supplementary Material 1 [file 12879_2025_11384_MOESM1_ESM.docx]

**Supplemental Material**

**Methods**

**Detailed inclusion and exclusion criteria**

CAP cohort

Children were enrolled into the CAP cohort if they presented to the emergency department (ED) with at least two signs and symptoms of CAP (i.e., age-adjusted tachypnea, nasal flaring, hypoxemia (<90% SpO_2_), rales or rhonchi, crackles, decreased breath sounds, lower chest indrawing, parent report of fever within 5 days of ED visit or current temperature of ≥ 39.0 °C) and either radiographic evidence of pneumonia, as read by the ED provider or on-call radiologist, or antibiotic prescription for suspected pneumonia. Children were excluded from the CAP cohort if they had received any dose of a pneumococcal vaccine within the 30-days prior to enrollment, had cystic fibrosis, presented with aspiration pneumonia, had a significant neurological disorder that may predispose the patient for aspiration pneumonia, were hospitalized within 30-days of the ED visit, transferred to the ED from an outside hospital within 48-hours of the ED visit, or were previously enrolled in any of the three cohorts.

URI cohort

Children were enrolled into the URI cohort if they presented to the ED with one or more signs and symptoms of URI (i.e., cough, sore throat, rhinorrhea/nasal congestion, ear pain or otitis media, parent report of fever within five days of the ED visit or current temperature of ≥39.0°C). Children were excluded from the URI cohort if they received any dose of a pneumococcal vaccine within 30-days prior to the ED visit, significant neurological disorders that may predispose the patient for aspiration pneumonia, were hospitalized in the previous 30-days of the ED visit or had a prior enrollment in any other cohort.

Control cohort

Children were enrolled into the control cohort if they presented to the ED, outpatient surgery, or a primary care practice for a non-infection-related medical complaint (e.g., well child check, tympanostomy). Children were excluded from the control cohort if they were suspected to have a respiratory infectious disease, evidence of concomitant infectious disease, known bronchial obstruction or a history of post-obstructive pneumonia, primary lung cancer or another malignancy metastatic to the lungs, present temperature of ≥ 39.0°C, significant immunosuppressive disorders (e.g., leukemia), receipt of any dose of pneumococcal vaccine within 30-days of the enrollment visit, and prior enrollment in any of the other cohorts.8,9

**Details on data collection elements**

The specific variables obtained by caregiver interview included child’s race and ethnicity, previous medical conditions, previous hospitalizations due to pneumonia, number of days of illness, and presence and days of the symptoms (i.e., fever, cough, difficulty breathing, fast breathing, vomiting, seizure, difficulty sleeping, decreased fluid intake, congestion, diarrhea, chest pain, abdominal pain, wheezing, and other non-listed symptoms). Information regarding medical care sought for the current illness was also obtained and any medications including corticosteroids or antibiotics that were prescribed in the past 14 days for the current or related illness (e.g., otitis media). Finally, parents were asked about their child’s influenza and COVID vaccination status during the season in which they were enrolled and. Notably, the COVID vaccination for children < 5 years was first available in June 2022 (>1 year into the enrollment of the study).

Data elements collected from the child’s electronic medical record included medications received (i.e., antibiotics and corticosteroid therapy), receipt and type of oxygen (i.e., nasal cannula/ aerosol mask, heated high flow nasal cannula, Continuous Positive Airway Pressure (CPAP)/ Bilevel Positive Airway Pressure (BiPAP) /Average Volume-Assured Support (AVAPS)), signs and symptoms (e.g., temperature at presentation, respiratory rate, heart rate, oxygen saturation (SpO2), wheezing, decreased breath sounds), overall general condition (i.e., alert, irritable, lethargic, unconscious), disposition if ED visit (i.e., discharged home or admitted), ED length of stay, and hospital length of stay. If the patient was admitted to the hospital, setting of admission was obtained (i.e., hospital medicine service, Intensive Care Unit (ICU), pulmonary, or other), antibiotics, and pneumonia-related complications (i.e., pneumothorax, empyema/pleural effusion, abscess, sepsis, pulmonary edema, necrotizing pneumonia, other). Pneumococcal vaccine was obtained via the Colorado Immunization Information System (CIIS), part of the Department of Public Health and Environment, an immunization record tracking system that follows a Colorado resident through life under the Colorado Registry Act of 2007. CIIS is integrated into the EHR and includes any immunization that a child received from any health care provider within the state and records vaccine exemptions for the child when applicable.

**Supplemental Table 1. Flow of the participants in each stage of recruitment by cohort**

|  | Controls | CAP | URI |
| --- | --- | --- | --- |
| Screened | 2745 | 734 | 906 |
| Eligible | 2167 | 521 | 583 |
| Approached | 1744 | 360 | 461 |
| Enrolled | 407 | 280 | 202 |

**Supplemental Table 2. Pneumococcal serotype detection by UAD assays among children with URI or CAP**

|  | **URI (N=202)** | **CAP (N=280)** |
| --- | --- | --- |
| UAD positive, n (%) | 10 (5) | 36 (13) |
| UAD-1 serotypes, n (%)^a^ |  |  |
| 1 | 1 (0.5) | 0 (0) |
| 4 | 0 (0) | 1 (0.4) |
| 5 | 0 (0) | 1 (0.4) |
| 6A/6C | 0 (0) | 2 (0.7) |
| 6B | 0 (0) | 2 (0.7) |
| 7F | 0 (0) | 2 (0.7) |
| 9V | 1 (0.5) | 0 (0) |
| 14 | 0 (0) | 5 (1.8) |
| 19A | 0 (0) | 1 (0.4) |
| 19F | 2 (1) | 6 (2.1) |
| 23F | 1 (0.5) | 1 (0.4) |
| UAD-2 serotypes, n (%) |  |  |
| 2 | 1 (0.5) | 2 (0.7) |
| 8 | 0 (0) | 1 (0.4) |
| 9N | 1 (0.5) | 6 (2.1) |
| 11A | 1 (0.5) | 0 (0) |
| 12F | 0 (0) | 1 (0.4) |
| 15B/15C | 2 (1) | 2 (0.7) |
| 17F | 2 (1) | 1 (0.4) |
| 20 | 1 (0.5) | 1 (0.4) |
| 22F | 1 (0.5) | 6 (2.1) |
| 33F | 0 (0) | 1 (0.4) |
| Abbreviations: CAP = community acquired pneumonia, UAD1 = urinary antigen detection assay 1 (for detection of serotypes 1, 3, 4, 5, 6A, 6B, 7F, 9V, 14, 18C, 19A, 19F, 23F), UAD2 = urinary antigen detection assay 2 (for detection of serotypes 2, 8, 9N, 10A, 11A, 12F, 15B, 17F, 20, 22F, 33F), URI = upper respiratory infection, URI = upper respiratory infection  ^a^ There were no UAD positive urine samples for serotypes 3, 10A, and 18C. | | |

**Supplemental Table 3.** **Pneumococcal Detection and Density by the Presence of a Respiratory Virus by Cohort**

|  | **URI (N=134)** | | | **CAP (N=189)** | | |
| --- | --- | --- | --- | --- | --- | --- |
|  | **With Virus (n=119)** | **Without Virus (n=15)** | **p-value^b^** | **With Virus (n=161)** | **Without Virus (n=28)** | **p-value^b^** |
| *S pneumoniae* positive nasal swab, n (%) | 32 (27) | 3 (20) | 0.76 | 51 (32) | 13 (46) | 0.14 |
| Nasal swab density log10 median (IQR) | 4.1  (3.2, 4.8) | 4.4  (3.7, 4.8) | 0.52 | 4.0  (3.1, 5.0) | 3.6  (3.4, 4.2) | 0.12 |
| Nasal swab density category (log10 density range)^c^, n (%) | | | | | | |
| High (4.44-5.99) | 11 (34) | 1 (33) | 1.00 | 19 (39) | 2 (15) | 0.19 |
| Medium (3.53-4.43) | 11 (34) | 2 (67) | 0.54 | 14 (29) | 5 (39) | 0.51 |
| Low (3.00-3.51) | 10 (31) | 0 (0) | 0.54 | 16 (33) | 6 (46) | 0.52 |
| Abbreviations: CAP = community acquired pneumonia, IQR = interquartile range, RPP = respiratory pathogen panel, URI = upper respiratory tract infection  ^a^ Among 22 control children who were tested by RPP, *S. pneumoniae* was not detected in any nasal swabs.  ^b^ The p-values were derived using Fisher’s exact test when comparing proportions and the median test when comparing the medians.  ^c^ The SP2020 rtPCR results (copies/mL) from positive nasal swabs were log10 transformed and categorized into one of three density tertile groups: low density (≤ 33%); medium density (34%-65%); high density (≥66%). | | | | | | |
